# The origin of circulating microbial DNA in the blood: where does it come from?

**DOI:** 10.1080/07853890.2025.2560605

**Published:** 2025-09-16

**Authors:** Zichen Zhang, Wenbo Ren, Yifei Wang, Taiyu Zhai, Jing Huang

**Affiliations:** a Department of Clinical Laboratory, The First Hospital of Jilin University, Changchun, China; b College of Medical Technology, Beihua University, Jilin, China

**Keywords:** Circulating microbial DNA, origin, membrane vesicles, blood, microorganisms

## Abstract

**Background:**

Circulating microbial DNA (cmDNA) is a significant biomarker involved in normal physiology, immunity, disease diagnosis, and pathogenesis. Its origin in peripheral blood has sparked extensive discussions and generated numerous hypotheses.

**Aim:**

This review explores four potential sources of cmDNA in the blood and analyzes research limitations and future prospects.

**Results:**

Four potential sources are collated and analyzed: exogenous microbes or DNA entering the peripheral blood after barrier damage; residual pathogen DNA remaining after infectious diseases; microbial translocation from sites such as the oral and intestinal mucosa into the bloodstream; and the extracellular vesicle delivery system, where microorganisms release vesicles to transfer DNA.

**Conclusions:**

The potential sources of cmDNA in the blood are not mutually exclusive and may be multifaceted, depending on an individual’s health condition, sampling time, and contamination control. In-depth study of these sources will open new avenues for early disease detection, precise diagnosis, and prognosis assessment, and encourage further exploration in this promising field.

## 1. Introduction

Circulating microbial DNA (cmDNA) represents a type of microbial nucleic acid component recently detected in circulation [1]. In healthy individuals, bacterial DNA constitutes the majority of blood cmDNA (over 95%), with the phyla *Pseudomonadota, Actinobacteriota, Bacillota,* and *Bacteroidota* as the core microbial cohorts [2–5]. A small fraction of blood cmDNA originates from eukaryotes and viruses [6]. Only 3% originates from eukaryotic sources, primarily from the fungal phyla *Basidiomycota* and *Ascomycota* [7]. while viral sources contribute approximately 0.4%, with non-pathogenic viruses such as human anelloviruses being predominant [8,9]. To a certain extent, the composition of cmDNA reflects the interactions between the host and microbial communities in various parts, collectively constituting a unique and dynamic reservoir of microbial genetic information within the human circulatory system [4]. Notably, cmDNA concentrations and compositions differ significantly between healthy individuals and patients, with alterations potentially linked to the progression of specific diseases [2]. For instance, distinctive patterns have been observed in the cmDNA of patients with diabetes mellitus and cardiovascular diseases, offering potential biomarker opportunities for early diagnosis and therapy [10,11]. Moreover, monitoring changes in blood cmDNA is important in cancer detection, prognosis, and therapy evaluation [12–15].

For the diagnosis and treatment of the aforementioned diseases, cmDNA has significant advantages compared to cytokines or other traditional clinical biomarkers. For instance, cmDNA detection is more convenient and can be analyzed rapidly using high-throughput sequencing technology [16]. Meanwhile, its source is specific, which provides more direct pathological information. It is noteworthy that the half-life of cmDNA is significantly shorter than that of traditional protein-based biomarkers, allowing its detection results to more sensitively reflect real-time disease dynamics, which makes it particularly suitable for immediate evaluation of treatment efficacy [17]. Additionally, extracellular vesicles, one of the sources of cmDNA, serve as natural carriers with the advantages of low cost, high stability, and ease of production, gradually emerging as alternatives to traditional methods of directly detecting cytokines by acting as natural carriers [2]. More importantly, the double-layered membrane structure of these vesicles effectively protects cmDNA from degradation by nucleases in the blood, extending the sample stability period to over 72h, significantly surpassing the preservation requirements for free DNA samples such as those used in PCR [18].

In recent years, the source of cmDNA in blood has piqued the profound interest of researchers in the field, prompting the formulation of numerous hypotheses regarding its genesis. Initial theories postulated that cmDNA enters the bloodstream following skin injuries caused by trauma, blood collection procedures, or surgical interventions, remaining as residual nucleic acid after immune clearance [4]. Alternative perspectives contended that cmDNA may originate from pathogens causing infectious diseases, either by direct invasion of the bloodstream or through disruption of the body’s endothelial and epithelial barriers [19]. Concurrently, a growing consensus among researchers favors the hypothesis of microbial translocation from other anatomical sites, suggesting that cmDNA originates from microbiota residing in regions such as the oral cavity or intestine [3,20–23]. Moreover, the most recent research has introduced a novel hypothesis, known as the extracellular vesicle delivery hypothesis, which postulates that microorganisms colonizing various body parts may release membrane-bound vesicles to transport their DNA *via* the circulatory system to the blood or other organs [2]. These diverse perspectives provide multifaceted insights into the origins of cmDNA in blood, further enhancing our comprehension and appreciation of this intriguing phenomenon [24].

Overall, the debate regarding the origin of cmDNA in blood remains unresolved, and a conclusive understanding is still elusive. Therefore, this review endeavors to assess the diverse hypotheses and perspectives pertaining to the potential sources of cmDNA in blood, thereby offering valuable insights and direction for future investigations into the origin of cmDNA and its clinical implications.

## 2. The development of blood cmDNA research

**Figure 1.**
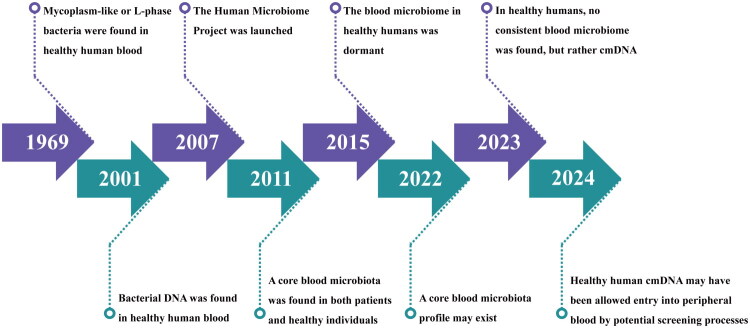
Key milestones in the evolution and advancement of blood microbiology research.

Blood microbiota is not a novel field of research. In fact, its study has undergone a protracted evolution (Figure 1). It began with Leeuwenhoek’s initial observation of the coexistence of red blood cells and bacteria in 1674 and was followed by reports in the mid-to-late twentieth century of bacteria being detected in patients’ blood, gradually gaining scientific recognition [25]. In 1969, Tedeschi broke new ground by reporting the presence of mycoplasma-like or L-form bacteria in the blood of healthy individuals [26]. This discovery was further validated in 1977, when researchers identified novel bacterial structures in both healthy and diseased human blood, affirming the existence of blood microbiota [27]. In 2001, Nikkari shed new light on the matter by pointing out the presence of bacterial DNA in healthy blood [28]. Shortly thereafter, McLaughlin reinforced these findings by employing multiple detection methods, including PCR amplification of 16S rRNA and gyrB genes, transmission electron microscopy (TEM), dark-field microscopy (DFM), and fluorescence *in situ* hybridization (FISH), all of which confirmed the presence of pleomorphic bacteria in healthy blood [29].

Subsequent to these initial discoveries, researchers have conducted extensive investigations into the origin and functions of these "mysterious visitors" found in the blood, leading to a proliferation of various hypotheses. In 2007, the launch of the Human Microbiome Project brought new momentum to the study of blood microbiota [30]. Scientists shifted their focus from analyzing individual microorganisms to conducting systematic research on the entire microbiota, aiming to unveil the mysteries of blood microbiota in a more comprehensive manner. In this process, Amar, J. proposed in 2011 that there exists a core microbiota in the blood, primarily composed of *Pseudomonadota* [31]. Until recent years, researchers have engaged in extensive debate and discussion on whether a core microbiome exists in the blood of healthy individuals. In 2022, Goraya observed differences in bacterial diversity among various blood components and noted similar trends across studies on blood microbiota, suggesting the possibility of a core blood microbiota spectrum independent of research environments or analytical methods [32]. However, in 2023, Tan et al. conducted a study to identify microorganisms in the peripheral blood of 8,892 healthy adults. After implementing a series of rigorous contamination exclusion procedures, they identified 117 types of microorganisms. These microorganisms were mainly associated with the commensal microbes in the gut, oral cavity, and urogenital tract. The study’s final conclusion challenged previous ones, proposing that there is no "core blood microbiome" in the peripheral blood of healthy individuals, but rather the presence of sporadic cmDNA [33]. Tan’s discovery seems to put an interim end to the debate regarding whether blood contains microbiota or cmDNA, but it also introduces new questions. Is it a microbiota or cmDNA that we detect in healthy blood? Do these microbes originate from endogenous blood pathways or exogenous invasions following bodily injury?

In addition, when comparing the studies by Goraya and Tan, there seems to be another issue that warrants attention: differences in detection methods. Goraya’s study utilized 16S rDNA, whereas Tan et al. employed metagenomic sequencing. It is currently uncertain whether this discrepancy is attributed to the detection methods. This is because 16S rDNA sequencing hinges on the amplification of specific primer pairs (such as the V3-V4 regions), and different primer combinations may exhibit biases towards certain microbial populations, leading to the inability to detect species that are not amplified. Therefore, the current detection methods for cmDNA are also an issue of concern. In Table 1, we have listed some representative past studies, along with their research methods and main conclusions.

**Table 1.** Summary of the process of methodological development in cmDNA research.

Sample Types	Detection Methods	Major Findings	Year	References
RBCs	Radiation uptake of nucleosides and amino acids by erythrocytes	Mycoplasma-like or L-phase bacteria may be found in the blood of healthy individuals.	1969	[26]
Blood	Filtered blood for bacterial culture	Novel bacterial structures in both healthy and diseased human blood.	1977	[27]
Blood	Real-time PCR methods with primers and a probe targeting conserved regions of the bacterial 16S rDNA gene	Seven phylogenetic groups and five bacterial divisions or subdivisions were identified in the blood.	2001	[28]
Blood	PCR amplification of 16S rDNA and gyrB genes and detected by microscopy (dark-field and transmission electron) and fluorescent *in situ* hybridization (FISH)	Polymorphic bacteria are present in the blood of healthy individuals, which exhibit limited growth and susceptibility to antibiotics. 16S rDNA gene sequencing showed that the bacterial genus with the closest homology was *Pseudomonadota*.	2002	[29]
Blood	PCR amplification of the 16S rRNA gene and Sanger sequencing	Bacteria that have been exclusively identified in clones include *Aquabacterium, Budvicia, Stenotrophomonas, Serratia, Bacillus,* and *Flavobacteria*.	2008	[34]
Blood	DNA was extracted from peripheral blood leucocytes,16S rDNA quantitative PCR, and pooled pyrosequencing	At the phylum level, *Pseudomonadota* represented 80% to 90% of all phyla in the blood, both in cases and controls. Within the *Pseudomonadota* phylum, at a genera level, the *Ralstonia* genus was the most prevalent.	2011	[31]
RBCs and Plasma	Blood suspensions are incubated on either trypticase soy blood agar (TSA) or blue lactose plates and are subsequently identified through colony PCR targeting the 16S RNA gene	Bacterial growth was observed in 35% of RBC fractions and 53% of plasma fractions. *Staphylococci, Propionibacterium, Micrococcus,* and *Bacillus* are most frequently found.	2015	[35]
Plasma	DNA-level analyses involved the amplification and sequencing of the 16S rRNA gene, RNA-level analyses were based upon the *de novo* assembly of unmapped mRNA reads and subsequent taxonomic identification, and classical microbiological culture	At the phylum level, the blood microbiome was dominated by *Pseudomonadota, Actinobacteriota, Bacillota,* and *Bacteroidota*. The key phyla detected were consistent regardless of which molecular method was used (DNA or RNA). Aerobic and anaerobic cultures were positive in eight out of the ten donor samples investigated.	2019	[4]
Plasma and blood	16S rRNA amplicon sequencing	Genus *Staphylococcus* and phylum *Chloroflexi* were increased in the blood of HE patients; conversely, an enrichment of species *Pseudomonas alcaligenes* was detected in the NHE group.	2020	[36]
Plasma	PCR amplification of the 16S rRNA gene sequencing	Circulating plasma microbiome profiles in patients with cirrhosis were distinct from those of the controls and were characterized by enrichment of *Comamonas, Cnuella, Dialister, and Escherichia.*	2022	[37]
Serum	Metagenome sequencing	Increased relative abundance of phyla *Actinobacteriota* and *Pseudomonadota*, decreased relative abundance of *Bacillota*, and reduced alpha diversity of the serum microbiome were associated with disease progression to esophageal adenocarcinoma.	2022	[12]
Plasma	Metagenomic next-generation sequencing	188 microorganisms were detected in 74.4% (96/129) patients, including viruses (58.0%), bacteria (34.1%), fungi (7.4%), and chlamydia (0.5%). Patients with acutely decompensated liver cirrhosis had a Non-Hepatotropic Virus (NHV) signature, and Cytomegalovirus (CMV) was the most frequent NHV. The NHV signature in acute-on-chronic liver failure (ACLF) patients was found to be similar to patients with sepsis and haematological malignancies. CMV was detected in 24.1% (14/58) of patients in the validation cohort.	2023	[38]
Blood	Metagenomic sequencing	117 microbial species were identified in 8892 samples, predominantly associated with commensal microorganisms from the gut, oral, and genitourinary tracts; no strong patterns of microbial co-occurrence or mutual exclusivity were observed, and no consistent associations between microbial presence and host phenotype were established, suggesting the absence of interacting microbial communities in healthy human blood.	2023	[33]
Blood	Blood culture was conducted using the BACTEC 9240 system, and PCR amplification of the 16S rDNA gene sequencing	The decreased abundance of anaerobic bacteria and the increased abundance of aerobic bacteria in blood samples contrasted with the predominantly anaerobic microbial flora structure in faecal samples. Moreover, the bacteria in the blood samples were predominantly Gram-negative, whereas those in the faecal samples were predominantly Gram-positive.	2024	[2]
Plasma	V3-V4 16S rRNA gene sequencing	*Pseudomonadota*, followed by *Bacteroidota*, *Bacillota,* and *Actinobacteriota*, were the dominant phyla in the cmDNA of all study subgroups.	2024	[39]

In our previous studies, based on a comparison of blood cmDNA and fecal microbiota information from the same cohort by 16S rDNA, we formulated a conjecture that the cmDNA in the peripheral blood of healthy individuals may enter the bloodstream after undergoing a potential screening mechanism [2]. However, it should be noted that this is merely an inference drawn after predicting the origin of cmDNA. To prove this conjecture, further extensive research is still required, such as analyzing changes in cmDNA following gut microbiota transplantation. It can thus be seen that, despite the current surge in research on cmDNA and the exciting progress achieved, there are still numerous issues that remain to be resolved. In Table 2, we have outlined the advancements and identified gaps in some typical studies conducted over the past five years.

**Table 2.** Summary of advances and gaps in cmDNA research over the past five years.

Study Population	Advances	Gaps	Year	References
Healthy subjects (*n* = 5) and asthmatic subjects (*n* = 5)	Further evidence for the concept of a core blood microbiome.	Small sample sizes	2019	[4]
20 rheumatoid arthritis patients, 4 ankylosing spondylitis patients, 4 psoriatic arthritis patients, and 4 healthy control subjects	The microbial community in Rheumatoid Arthritis differs from that in Ankylosing Spondylitis, Psoriatic Arthritis, and healthy states, and normalises in some patients after treatment, additionally demonstrating the presence of a blood microbiome in the healthy population.	Sample cohorts were procured from different sources	2019	[40]
215 serum samples (50 healthy controls (HC), 23 atypical hyperplasia (AH), 34 chronic gastritis (CG), 108 gastric cancer (GC))	The bacterial communities in HC, CG, AH, and GC groups were structurally different, with alpha diversity (OTU numbers) decreasing from HC to GC. Illustrated the characteristics of the serum microbiome composition in patients with GC.	Could not determine whether these microbial changes were a cause or consequence of GC progression	2019	[41]
103 non-MI individuals and 99 MI patients	In MI patients, lower cholesterol-degrading bacteria and increased 16S rDNA concentration were linked to blood cholesterol levels, and the Caulobacterales order and Caulobacteraceae family were both considerably reduced.	Failure to focus on the dynamic development of cmDNA from the pre-disease stage to the disease stage	2019	[42]
30 liver cirrhosis patients with hepatic encephalopathy (LC-HE), 33 liver cirrhosis patients with non-hepatic encephalopathy (LC-NHE), and 26 healthy controls	Genus *Staphylococcus* and phylum *Chloroflexi* were increased in the blood of HE patients, conversely, an enrichment of species *Pseudomonas alcaligenes* was detected in the NHE group.	Restricted Group Segmentation in Patient Studies	2020	[36]
25 colorectal cancer (CRC) patients, 10 colorectal adenoma (CRA) patients, and 22 healthy controls (HC)	Alterations of circulating bacterial DNA have been demonstrated in colorectal neoplasia patients. Additionally, species diversity in HC was slightly higher compared with that in CRC/CRA patients.	Failure to focus on the dynamic development of cmDNA from the pre-disease stage to the disease stage	2021	[43]
69 patients (depressive disorder or bipolar disorder)	This study was the first to compare the serum microbial DNA composition and psychiatric symptom severities in mood disorders.	In a cross-sectional study, causality could not be determined	2021	[44]
Subjects undergoing bariatric surgery were recruited into sex- and BMI-matched subgroups with T2D (*n* = 24) or without T2D (*n* = 24)	The circulating bacterial signature reflects metabolic disease and its improvement after bariatric surgery, and provides evidence, taking into account potential contaminants, for the presence of living bacteria in the blood.	Cannot fully exclude contamination *via* puncture of the skin or other environmental sources	2021	[45]
81 serum specimens collected across the esophageal adenocarcinoma	Firstly describes unique blood-based microbial profiles in patients across EAC carcinogenesis, which are further utilised to establish a novel circulating diagnostic and prognostic metagenomic signature for EAC.	No strict controls and stratification in the patient profile that might confound the microbiome profile of each patient.	2022	[12]
129 (study cohort) and 58 (validation cohort) acutely decompensated liver cirrhosis patients, and 120 controls (10 healthy volunteers, 20 stable cirrhosis, 39 severe, and 81 hematological malignancies)	188 microorganisms were detected in 74.4% (96/129) patients, including viruses (58.0%), bacteria (34.1%), fungi (7.4%), and chlamydia (0.5%). Patients with acutely decompensated liver cirrhosis had an NHV signature, and CMV was the most frequent NHV. The NHV signature in ACLF patients was found to be similar to patients with sepsis and haematological malignancies. CMV was detected in 24.1% (14/58) of patients in the validation cohort.	Untargeted plasma mNGS testing is not truly comprehensive, as this study focused on microbial cfDNA and RNA viruses, such as hepatitis C and E viruses, which were not included	2022	[38]
28 liver disease patients (14 ALD patients and 14 NAFLD patients) and 8 healthy controls.	ALD patients showed a significant but temporary increase in microbial DNA quantity in the hepatic and systemic venous blood because of alcohol intervention. However, the intervention did not cause a significant change in microbial DNA quantity in the hepatic and systemic venous blood in the healthy controls and NAFLD patients.	Small sample sizes	2024	[46]
Healthy subjects (*n* = 26)	Blood, faecal, and urine samples from the same participants were also tested.	Small sample sizes	2024	[2]
251 lung cancer patients, 165 healthy controls	The cmDNA profiles of lung cancer patients exhibit unique characteristics. Furthermore, a cmDNA-based diagnostic model of lung cancer has been developed, which exhibits high accuracy in identifying the disease. Importantly, cmDNA analysis can also predict lung cancer recurrence after surgery, providing valuable information for patient management and prognosis.	Further research necessitates a larger sample size of tumor tissues	2024	[14]
Advanced melanoma patients (*n* = 66) before and during the anti-PD-1 therapy (approximately 3 and 12 months after the start)	This study demonstrated the association between circulating cmDNA signatures, plasma sCD14 concentration, and clinical outcomes in advanced melanoma patients undergoing anti-PD-1 therapy.	Marker gene sequencing results will not include bacterial/archaeal sequences unless they cover the amplified region of the targeted gene, such as the V3-V4 hypervariable region of the 16S rRNA gene	2024	[39]
Colorectal cancer patients (*n* = 75) and healthy individuals (*n* = 25)	High 16S rRNA and *E. coli* detections were observed in all patients and controls. Only the detection of *F. nucleatum* was significantly higher in metastatic non-excised CRC, compared to controls (*p* < 0.001), non-metastatic excised CRC (*p* = 0.023), and metastatic excised CRC (*p* = 0.023).	The lack of thorough examinations of detailed blood microbiota profiling among participants	2024	[47]

## 3. Primary hypotheses of the origin of cmDNA in blood

### 3.1. Exogenous microbes or DNA entering the peripheral blood

Regarding the origin of cmDNA in blood, initial perspectives suggested that the presence of cmDNA observed in the blood of healthy individuals might be attributed to the exogenous introduction of microbes or DNA following invasive medical procedures or tissue injuries. Numerous invasive medical procedures can lead to the entry of foreign microbes or DNA into the peripheral blood. For instance, non-surgical interventions (such as catheter insertion, intravenous infusions, and hemodialysis) and common medical procedures (like punctures and surgeries) can elevate the levels of bacteria and their metabolic products in the blood, subsequently activating the systemic immune system and maybe increasing the levels of cmDNA in the blood [3,48,49]. In addition, in the process of gene therapy, Adenovirus and Lentivirus, as commonly used viral vectors, can carry exogenous DNA into host cells. In the design and application of these vectors, exogenous DNA is usually introduced at the stage of gene delivery and integration to repair or replace defective genes. However, during this process, viral vectors may also carry non-target DNA fragments, which may include potential microbial DNA [50].

Furthermore, exogenous microbes or DNA may also enter the bloodstream through trauma. When the body sustains injuries such as insect bites, animal bites or scratches, and mechanical injuries from external forces, the skin barrier is disrupted, allowing external microbes to invade the blood, resulting in the presence of microbial DNA in human blood [20]. Additionally, when organs like the liver and kidneys experience dysfunction or damage, they may affect the homeostasis of the human microbiota, thereby influencing the species diversity and relative abundance of microbial communities in blood cmDNA [51].

Analysis of the species composition of skin and blood microbial communities, through comparison of skin and blood microbiota, revealed that *Cutibacterium acnes* was the predominant microorganism in both the microbiota of easily damaged dry skin (the palmar forearm) and the blood [33,52]. This suggests that cmDNA in the blood may result from exogenous microbial invasion following tissue damage. Usually, live microorganisms entering the bloodstream can trigger an immune response in the body and lead to infections, such as surgical wound infections or HIV infections resulting from blood transfusions [53,54]. However, recent studies have increasingly demonstrated that even after rigorous decontamination analyses, low-biomass cmDNA can be detected in the blood of healthy individuals, and it persists [55]. Furthermore, there is evidence that some microorganisms enter the blood in a dormant state and do not elicit an immune response in the host [56]. These findings suggest that cmDNA can stably exist in the blood and is not solely attributable to exogenous contamination. Therefore, although the potential endogenous origin of cmDNA warrants further investigation, exogenous microbial DNA invading the bloodstream following skin or tissue mucosal barrier compromise and immune clearance is one of the potential sources of blood cmDNA.

### 3.2. Residuals after infectious diseases

Infectious diseases are also considered an important source of cmDNA in the blood. For instance, Cytomegalovirus (CMV) is a well-established potential risk during blood transfusions and organ transplantations. As a commonly latent infectious agent, CMV can be transmitted to recipients *via* blood or transplanted organs [57]. Research has shown that CMV DNA can be transmitted through blood transfusions, particularly in immunocompromised recipients, resulting in latent infection [58]. In the context of organ transplantation, donor organs harboring latent CMV infections may transmit viral DNA to recipients, leading to CMV reactivation. Consequently, CMV DNA may be detected in the blood of recipients after transplantation [59].

In some other infectious diseases that involve live microorganisms, pathogens may spread throughout the body *via* the bloodstream, potentially leading to bacteremia or septicemia [60]. Furthermore, the inflammatory state caused by infection triggers the activation of immune cells and cellular damage within the body, further contributing to changes in the level of cmDNA in the blood. Studies have demonstrated significant differences in the concentration and composition of cmDNA between patients and healthy individuals. For instance, in healthy individuals, the abundance of *Actinobacteriota* decreases in patients with septicemia [61]. In patients with severe acute pancreatitis (SAP), the abundance of *Bacteroidota* and *Bacillota* increases, while the abundance of *Actinobacteriota* decreases [62]. Additionally, the levels of cmDNA are significantly elevated in patients with septicemia, severe pneumonia, inflammatory bowel disease (IBD), and human immunodeficiency virus (HIV) [63–65]. However, the biodiversity of IBD and HIV patients is significantly reduced [65]. In these disease states, cmDNA may function as pathogen-associated molecular patterns (PAMPs), activating pattern recognition receptors (PRRs) on immune cells, such as Toll-like receptor 9 (TLR9). This activation triggers the release of inflammatory cytokines, subsequently leading to the degradation of tight junction proteins between epithelial and endothelial cells, thereby increasing cellular permeability [66,67]. A recent study on HIV has lent support to this hypothesis by highlighting the potential significance of epithelial and endothelial cell-mediated increased paracellular permeability in enabling bacterial translocation into the bloodstream [68].

However, Marnie Potgieter et al. proposed a groundbreaking theory that microorganisms in the blood of healthy individuals may exist in a dormant or non-immediately culturable state [56]. This theory challenges the conventional view that blood cmDNA or microbes are solely linked to infectious diseases, thereby paving a new path for blood microbiology research. This clue indirectly proves that blood cmDNA is not necessarily derived exclusively from infectious disease residues. Its origin needs to be considered in the light of other factors. Additionally, we need to consider other factors, such as the genuine causal relationship between blood cmDNA and diseases, which will provide a solid theoretical foundation for future experimental research and clinical validation.

### 3.3. Microbial translocation

As research progresses, it has become increasingly evident that the presence of microbes in the blood is largely attributed to microbial translocation from body sites enriched with microbiota [22]. This translocation involves the migration of microorganisms or their components, such as lipopolysaccharides (LPS), peptidoglycan, and DNA, from the human microbiota into the bloodstream through epithelial mucosa in organs that interface with the external environment [60]. In certain pathological states, such as intestinal barrier dysfunction or immune system impairment, microbes can migrate to the blood and release DNA.

Regarding microbial translocation, numerous studies have provided us with various perspectives. For instance, in 2005, Esther Jiménez detected the presence of microorganisms in the umbilical cord blood of newborns [69]. Subsequently, in 2013, Lisa J. Funkhouser proposed an intriguing viewpoint that microorganisms might enter the infant’s bloodstream before birth through maternal-fetal transmission [70]. In 2025, research by Wenjia Wang indicated that metabolites associated with the infant microbiome are provided by the mother and vertically transmitted to the infant. These studies have provided new clues for explaining the origin of microorganisms in infant blood [71]. However, the concept of maternal-fetal transmission of microorganisms is currently viewed with a high degree of skepticism by the scientific community, due to the use of molecular approaches with an insufficient detection limit to study "low-biomass" microbial populations, the lack of appropriate controls for contamination, and the fact that the reliable ability to derive axenic animals *via* cesarean sections strongly supports the sterility of the fetal environment in mammals [72,73]. Despite the significant controversies surrounding the mechanisms of microbial information transfer between mothers and infants, it is difficult for us to simply overlook or rule out the possibility of microbial transmission between mothers and infants.

The following evidence may offer indirect support for our aforementioned inference. In 2008, A.L. Cogen et al. proposed that skin microbiota can escape into the bloodstream [20]. The following year, Iwai T et al. hypothesized that blood microbes may originate from the oral cavity [21]. More recently, in 2019, Emma Whittle et al. conducted a comparative analysis of data from the Human Microbiome Project and healthy human blood microbiota, further confirming that blood microbes exhibit the closest resemblance to those found in the skin and oral cavity [4]. These studies have not only validated previously proposed hypotheses but also revealed the complex and intimate connections among microbial communities throughout the human body. The microbial populations in different body parts do not exist in isolation; rather, they constitute an interconnected and dynamically changing system. The mechanisms of microbial migration and communication offer us a new perspective for understanding the overall functions of the human microbiome.

Certainly, while the hypothesis of microbial translocation has gained widespread acceptance among researchers, it continues to be a subject of debate. Previously, it was commonly believed that bacteria could only invade the bloodstream if the epithelial mucosal barrier was compromised. However, with advancing research, we have uncovered that bacteria can gain access to the circulatory system through various mechanisms, even when the intestinal mucosa remains intact [74].

For instance, the sampling action role of antigen-presenting cells (APCs) at mucosal surfaces exemplifies this process. These cells, such as dendritic cells and macrophages, are capable of capturing bacterial DNA antigens on the mucosal surface and transporting them into the bloodstream to elicit an immune response [75]. Goblet cells, which secrete intestinal mucus, and mucosal lymphocytes (specialized epithelial cells of mucosa-associated lymphoid tissues) located over Peyer’s patches, also serve as microbial carriers that facilitate the transit of microorganisms from the intestine to the blood circulation [32]. This revelation has opened up a new understanding of how bacteria can enter the bloodstream. Building on this discovery, in 2017, Kowarsky et al. put forth an intriguing proposal: the circulating cell-free DNA found in the blood may originate from novel, understudied bacteria and viruses within the human intestinal microbiota [3]. This provides a novel perspective on the sources of blood microbiota. Subsequently, in 2019, Diego J. Castillo advanced a hypothesis suggesting that the microbiota present in the blood could originate from intestinal microbial translocation [23]. While this theory holds some logical merit, our research has demonstrated that there are significant differences in bacterial diversity between healthy intestinal microbiota and healthy blood microbiota. Specifically, the intestinal microbiota is typically dominated by *Bacillota* and *Bacteroidota*, whereas the blood microbiota is primarily composed of *Pseudomonadota* [48]. These findings have prompted a reevaluation of the generalizability and applicability of the microbial translocation hypothesis.

Our team has recently undertaken an in-depth comparative analysis of the microbial compositions in blood and stool samples from the same research cohort. Building upon the methods employed in the study by Nejman et al. to exclude information on conventional contaminants [76]. We additionally collected cotton swab samples from the skin, urethral orifice, and anal surface of volunteers. The microbial information detected from these samples was further excluded as potential contamination information. Our findings indicate that the abundance of anaerobes in blood samples is notably lower, while the prevalence of aerobes and facultative anaerobes is comparatively higher [2]. This stands in stark contrast to the anaerobic-dominated flora structure typically observed in stool samples. Additionally, we have discovered that bacteria in blood samples are predominantly Gram-negative, in stark contrast to the primarily Gram-positive bacteria found in stool samples. Notably, the cmDNA in blood samples exhibits unique characteristics, including markers associated with mobile genetic elements, biofilm formation, potential pathological conditions, and stress tolerance, which are rarely detected in stool samples [2].

Drawing upon the perspectives of Tan et al. and our team’s latest research, we further hypothesize that while the cmDNA in the bloodstream is likely sourced from microbial colonization sites such as the intestine or mouth, its translocation into the blood is not a random process [2,33]. Rather, it seems to be the outcome of a potential specific mechanism of filtration. However, it is worth noting that current research has yet to elucidate precisely how the intestinal or oral mucosal barriers contribute to this potential filtration mechanism of cmDNA, resulting in significant disparities in microbial information between blood cmDNA and fecal matter. Such changes in the host’s lifestyle (including living environment, living habits, and dietary intake habits) may be potential factors contributing to alterations in cmDNA. Lack of exercise or poor dietary habits may lead to gut barrier dysfunction or impaired immune system function, thereby increasing the likelihood of microbial translocation or DNA migration from the gut, oral cavity, or other sites into the bloodstream, subsequently causing changes in cmDNA [77]. Therefore, while the translocation theory provides a reasonable explanation for the origin of cmDNA, it is clearly influenced by many other factors or underlying mechanisms.

### 3.4. Extracellular vesicle delivery system

Recent studies have revealed that, in addition to previously mentioned mechanisms, the diverse extracellular vesicles produced by microorganisms appear to offer a fresh perspective on our specific experimental outcomes [78,79]. These vesicles, known collectively as membrane vesicles (MVs), range in diameter from 20 to 400 nanometers. These tiny vesicles play crucial roles in various biological processes within the human body, including DNA transfer, transportation of virulence factors, interception of bacteriophages, antibiotics, and eukaryotic host defense factors, cell lysis, export of cellular metabolites, and intercellular communication [80,81].

Notably, microbial MVs exhibit immunomodulatory activities, indicating their potential as vaccine candidates, and demonstrating significant promise in anticancer drug delivery and nanotechnology applications [81]. The presence of these vesicles allows microbiota colonizing the gut or oral cavity to deliver their genetic material into the bloodstream in a non-cell-dependent manner, without eliciting an immune response from the host. Recent research has further uncovered that these vesicles are, in fact, the primary mechanism bacteria utilize to deliver various hydrophobic compounds to the host under normal conditions [80,81]. This revelation provides us with a unique understanding of the interactions between microorganisms and their hosts.

It is worth noting that MVs produced by diverse microorganisms exhibit remarkable differences in their uptake mechanisms, surface compositions, and cargoes, enabling them to possess unique properties and targeted functions in genetic material transmission [82]. Studies suggest that intestinal microbiota-derived MVs are internalized by intestinal epithelial cells *via* endocytosis, subsequently crossing the endothelial barrier to enter the bloodstream and disseminate to various organs throughout the body [83]. Our research reveals that cmDNA in blood primarily mirrors the presence of Gram-negative bacteria, whereas fecal cmDNA predominantly reflects Gram-positive bacteria [2]. This discrepancy likely stems from the distinct surface compositions, cargoes, and uptake mechanisms of MVs from Gram-positive and Gram-negative bacteria.

Endocytosis serves as the primary mechanism for mammalian cells to internalize MVs from Gram-negative bacteria, yet the uptake process is influenced by the surface properties and carriers of various MVs. Extensive research has uncovered the internalization mechanisms and distant communication capabilities of *Bacteroides thetaiotaomicron*-derived bacterial MVs [84]. Conversely, there is a paucity of studies on the internalization pathways of MVs from Gram-positive bacteria. Current research indicates that MVs from Gram-positive bacteria primarily enter cells *via* a clathrin-mediated pathway, albeit with a relatively slower uptake kinetics [85].

Additionally, there are significant disparities in the surface compositions of MVs from Gram-negative and Gram-positive bacteria. For instance, MVs from Gram-negative bacteria are abundant in LPS on their surfaces, whereas MVs from Gram-positive bacteria are devoid of LPS [83]. This variance could influence the interaction between MVs and intestinal epithelial cells, thereby affecting the internalization and trafficking of these vesicles. Because LPS can enhance intestinal permeability, it facilitates the passage of LPS-containing gram-negative bacterial MVs through the intestinal barrier into the blood circulation [86]. These surface disparities likely play a pivotal role in regulating the selectivity and trafficking of vesicles by intestinal epithelial cells.

**Figure 2.**
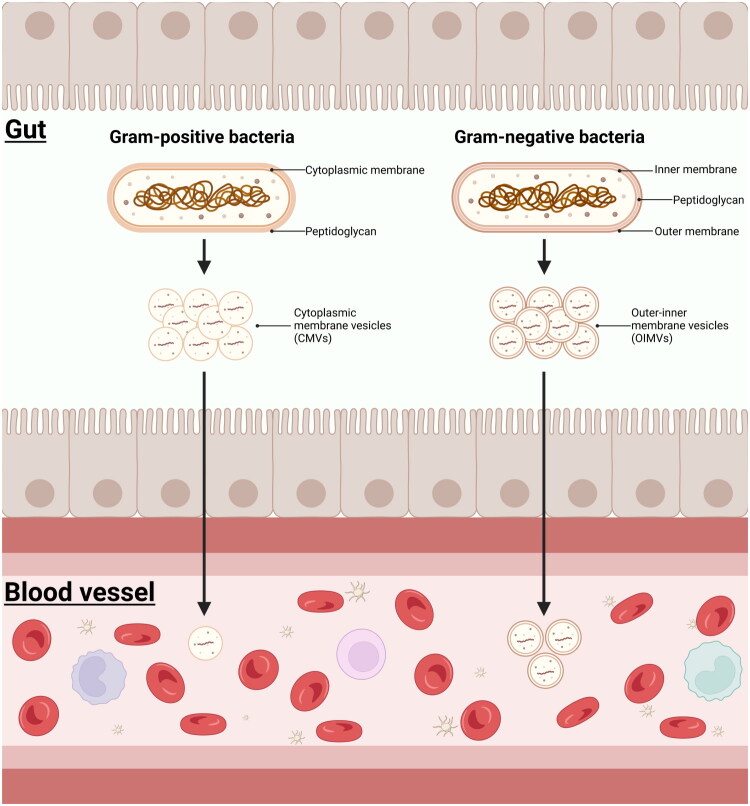
Extracellular vesicle delivery mechanism. This figure has been licensed for publication by biorender [created in BioRender. Zhai, T. (2025) https://BioRender.com/yiijq7c].

Although this perspective is currently grounded in existing evidence, it serves as a plausible hypothesis regarding the origin of blood cmDNA (Figure 2). However, to elucidate the precise source and functional mechanisms of blood cmDNA, further investigation is imperative.

## 4. Limitations of the current studies

In the field of cmDNA research, contamination control represents one of the major obstacles hindering progress. Contamination can occur at various stages of experimentation, including sampling, the laboratory environment, operator handling, use of reagents and consumables, as well as cross-contamination between samples or during sequencing processes [87]. These contaminants can severely impact the accuracy and reliability of experimental results. To address these challenges, researchers have implemented a series of measures to reduce the risk of contamination. Physical protective measures, such as cleaning the skin before sampling, using UV lights to disinfect biosafety cabinets, and requiring laboratory personnel to wear disposable gloves and surgical gowns, aim to minimize contamination arising from physical contact during experiments [2,10,55]. Furthermore, transferring initially collected samples into individual tubes for independent analysis can effectively reduce the risk of cross-contamination between samples [4]. In recent years, bioinformatics methods have also played a significant role in contamination exclusion, with various filtering methods developed by Tan et al, for example, aiding researchers in identifying and excluding contamination during data analysis [33]. Although these methods have improved the contamination issue in cmDNA research to some extent, ongoing efforts and innovations are still required. A multi-layered approach combining physical protection, bioinformatics, and improvements in laboratory operations is an effective strategy to tackle this problem. Additionally, establishing detailed standard operating procedures (SOPs) and contamination control guidelines will assist researchers in applying best practices across different laboratory conditions, thereby enhancing the reproducibility and reliability of research findings.

In studies of cmDNA, comparing results obtained from different detection methods frequently encounters various limitations. These limitations not only impair the comparability of results but also have the potential to cause misunderstandings and incorrect conclusions. For example, disparities in sequencing methods can give rise to variations in sequencing depth and the detectable scope of microbial information (Table 1). Based on the limitations of existing detection methods, recent research has revealed a bidirectional regulatory relationship between the host’s biological clock and the rhythm of the microbial community [88]. This perspective indicates that different sampling times can influence the determination of cmDNA sources. Since the composition and activity of the microbial community vary with the host’s circadian rhythm, samples collected at different times of the day may exhibit different microbial community characteristics, thereby affecting the cmDNA analysis. Therefore, caution must be exercised regarding such technical differences when concluding horizontal comparisons and drawing conclusions in similar contexts. To enhance result reliability, researchers should carefully consider various variables in experimental design, including sampling time, sample processing, sequencing platform selection, and data analysis methods. Furthermore, establishing standardized experimental and analytical protocols, along with the sharing of data and methods across different studies, can help alleviate the impact of these limitations. This, consequently, facilitates the drawing of more reliable and consistent conclusions under identical conditions.

In addition, it is imperative to pay attention to the dynamic changes in cmDNA from the pre-disease stage to the disease stage. For instance, a study collected samples from patients at different stages of gastric cancer progression and categorized them into control, atypical hyperplasia, chronic gastritis, and gastric cancer groups. The results revealed that there were differences in the bacterial community structure across the groups, with the α-diversity (i.e. the number of operational taxonomic units, or OTUs) progressively decreasing from the control group to the gastric cancer group [41]. This suggests that it is necessary to shift from simple associations between microorganisms and diseases to the analysis of causal mechanisms and dynamic processes. Further integration of multi-omics data analysis can systematically reveal how microorganisms influence host immune or metabolic functions by releasing specific DNA fragments.

The limitations of current cmDNA research are multifaceted. Firstly, contamination control remains a significant challenge, as contamination can occur at multiple experimental stages, and despite measures taken, further efforts are still needed. Secondly, when comparing results obtained using different detection methods, limitations such as differences in sequencing methods and the influence of the host’s biological clock on the rhythm of the microbial community compromise the comparability of results. Finally, understanding the dynamic changes in cmDNA from the pre-disease stage to the disease stage is also crucial. Addressing these limitations through the adoption of SOPs, standardized protocols, and multi-omics data analysis is vital for advancing cmDNA research and ensuring the accuracy and comparability of results.

## 5. Conclusions and future prospects

**Figure 3.**
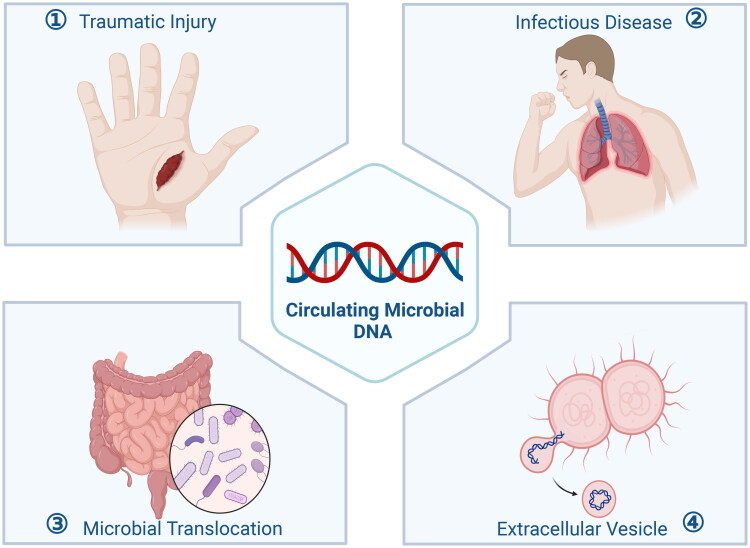
Four potential origins of blood cmDNA. This figure has been licensed for publication by biorender [created in BioRender. Zhai, T. (2025) https://BioRender.com/g26v408].

The sources of cmDNA in the blood are complex and mainly include direct microbial invasion, residual DNA after infection, migration of microorganisms from other colonization sites, or transportation *via* pathways such as EVs. Among these, EVs have become a research hotspot because they can efficiently carry DNA, RNA, and proteins, thereby influencing host cell functions (Figure 3). These sources are not mutually exclusive; instead, they may synergize to jointly determine the composition and characteristics of cmDNA.

Elucidating the origins and action mechanisms of cmDNA presents significant challenges, primarily arising from the technical difficulties inherent in research, such as limitations in sample size and interference from potential contaminants. Future research should concentrate on two key directions: first, precisely differentiating between the exogenous (microbial-derived) and endogenous (host-derived) origins of cmDNA; second, conducting large-scale longitudinal studies with dynamic analysis through long-term, timed sampling from diverse populations. Long-term sampling aids in identifying key factors influencing fluctuations in cmDNA levels, while timed sampling can prevent detection biases in species composition caused by temporal variations [88]. More critically, research needs to investigate in depth whether specific microorganisms are the primary contributors to cmDNA and whether these microorganisms employ intestinal selective mechanisms to transport cmDNA into the bloodstream. These explorations will not only enhance our understanding of the role of cmDNA in health and disease but also potentially unveil its substantial potential as a disease biomarker, providing a basis for the development of novel diagnostic tools.

The research on cmDNA holds vast potential for clinical applications. By monitoring the presence and fluctuations of specific microbial DNA in the blood, early disease warning can be realized. In the realm of personalized medicine, long-term tracking of cmDNA dynamics can uncover new therapeutic targets. Taking fecal microbiota transplantation (FMT) as an example, highly sensitive detection techniques enable real-time monitoring of changes in the recipient’s cmDNA, facilitating the assessment of the colonization effectiveness of transplanted microorganisms and the repair status of the intestinal barrier. This, in turn, upgrades the traditional "one-size-fits-all" approach to a companion-style personalized management model. Moreover, by integrating artificial intelligence to analyze individualized cmDNA data, it becomes feasible to predict health risks in real time, detect antibiotic resistance genes and probiotic expression levels, and dynamically optimize drug combinations or microbial intervention strategies, ultimately achieving full-cycle precision management from disease warning to prognosis.

To ensure the effectiveness and reliability of cmDNA in clinical applications, standardized research protocols and stringent contamination control measures will be essential. These efforts will significantly enhance the reproducibility and credibility of research results, paving the way for clinical applications. As the aforementioned research directions progress in the future, we will better elucidate the complex sources of cmDNA and its crucial role in human health, ultimately providing innovative strategies for disease diagnosis, prevention, and treatment.

## Data Availability

Data sharing is not applicable to this article as no new data were created or analyzed in this study.
